# Anal Squamous Cell Carcinoma with Bilateral Renal Metastases: A Rare Presentation with Literature Review

**DOI:** 10.3390/curroncol33010064

**Published:** 2026-01-22

**Authors:** Khujasta Gul, Saivaishnavi Kamatham, Guido Chiriboga, Ahmed Abdelhakeem, Aziza Nassar, Conor O’Donnell, Umair Majeed

**Affiliations:** 1 Department of Hematology/Oncology, Mayo Clinic Florida, Jacksonville, FL 32224, USA; gul.khujasta@mayo.edu (K.G.); kamatham.saivaishnavi@mayo.edu (S.K.); abdelhakeem.ahmed@mayo.edu (A.A.); odonnell.conor@mayo.edu (C.O.); 2 Mayo Clinic Alix School of Medicine, Jacksonville, FL 32224, USA; chiriboga.guido@mayo.edu; 3 Department of Pathology, Mayo Clinic Florida, Jacksonville, FL 32224, USA; nassar.aziza@mayo.edu

**Keywords:** anal squamous cell carcinoma, bilateral renal metastases, rare metastasis, chemoradiotherapy, immunotherapy

## Abstract

Anal squamous cell carcinoma (ASCC) is a rare malignancy of the lower gastrointestinal tract, with distant metastases typically involving the liver and lungs. Metastasis to the kidneys is uncommon, and only one prior case has been reported in the literature. Herein, we report a rare presentation of a patient with biopsy-confirmed metastatic ASCC presenting as bilateral renal lesions. We then provide a review of the literature for rare metastatic presentations of ASCC, highlighting all the cases described in the literature. Clinicians should maintain a high index of suspicion for unusual metastatic sites, perform targeted imaging and biopsy when indicated, and consider systemic therapies to optimize outcomes in rare metastatic presentations.

## Simple Summary

While anal squamous cell carcinoma typically presents with metastases to the liver or lungs, rare metastases sites have been reported. Here, we present a case of rare metastasis to both kidneys and review other cases of rare metastatic sites available in the literature. These cases should serve as reminders that cancers, including anal squamous carcinoma, can present in an atypical manner and increased awareness of these variations is warranted.

## 1. Introduction

Anal cancer is a rare malignancy. Among anal cancers, anal squamous cell carcinoma (ASCC) is the most common histological subtype. Anal cancer accounts for 2% of all the gastrointestinal tract malignancies [1,2,3]. The annual incidence of anal cancer is one to two cases per 100,000 individuals, with recent years showing an increase of nearly 2.2% per year in Western countries [4]. In 2025, an estimated 10,930 new cases of anal cancer and 2030 deaths are projected for this disease [5]. Human papillomavirus (HPV) is a key risk factor with 80–85% of anal cancer cases associated with HPV 16 infection. Other risk factors for include unprotected anal sexual activity, men who have sex with men (MSM), a prior history of gynecological malignancy in women, and human immunodeficiency virus (HIV) infection, individuals with solid organ transplants, autoimmune disorders, old age, female sex and smoking [3]. Rectal bleeding is the most common symptom, while other symptoms might include changes in stool frequency, pain or fullness in the area, itching and a lump or a mass at the anal opening [6]. Standard care for localized or locally advanced ASCC involves concurrent chemo radiation therapy (CRT) based on the Nigro protocol, which was initially developed in the 1970s. Since then, the use of definitive CRT using 5FU/capecitabine and mitomycin as an organ-preservation approach has been confirmed as the standard of care in several phase III randomized studies [7]. About 30% of patients with ASCC develop locoregional failure, leading to significant morbidity as well as a risk of distant recurrence and mortality [2]. Locoregional relapses may be treated with salvage abdominal perineal resection (APR). However, 10–20% of patients experience distant relapse after definitive CRT and about 10% of patients present with de novo metastatic disease [8]. The most common distant metastatic sites of ASCC include the liver and lungs [9,10]. Unusual metastatic sites have also been documented in the literature, including the iris and cauda equina [11,12]. ASCC metastases are heterogeneous, and emerging evidence indicates that a subset of patients with oligometastatic disease may benefit from a multimodal strategy that includes metastasis-directed therapy with curative intent [13]. Once CRT achieves local tumor control, metastasis-directed interventions such as surgical resection, stereotactic body radiotherapy (SBRT), or other ablative techniques can be considered. The standard of care for metastatic disease includes carboplatin and paclitaxel based on the phase 2 InterAAct trial [14]. A recently published PODIUM-303/InterAACT-2 trial showed the benefit of the PDL1 agent Retifanlimab to carboplatin and paclitaxel with improvement in overall survival (OS) [15]. Here, we present an exceptionally rare presentation and management of metastatic ASCC with bilateral renal metastases. We then review the available literature for other rare sites of metastatic involvement in this cancer and subsequent management so that this article can serve as a review for all such cases in the literature and to increase the awareness of atypical metastatic patterns and management.

## 2. Detailed Case Description

A 43-year-old male without any comorbid conditions was diagnosed with anal squamous cell carcinoma after he presented with anal discomfort and bright red blood per rectum. A colonoscopy and biopsy confirmed the diagnosis along with the presence of human papilloma virus (HPV) infection. Human immunodeficiency virus (HIV) testing was negative, and staging workup did not reveal any evidence of metastatic disease.

The patient was treated with concurrent chemoradiotherapy (CRT) with 5-fluorouracil and mitomycin which he completed in June 2022. The patient had a complete response to chemoradiotherapy with no evidence of specific mass on CT abdomen/pelvis and no evidence of malignancy on flexible sigmoidoscopy. Six months post-CRT, he started experiencing rectal pain, and repeat sigmoidoscopy confirmed recurrent anal cancer. Restaging scans showed locoregional progression of disease with the mass involving the internal anal sphincter along with the involvement of the bilateral inguinal lymph node. Subsequently, the patient underwent salvage abdominopelvic resection and bilateral inguinal lymphadenectomy. Pathology revealed a 2.3 cm residual poorly differentiated SCC; surgical margins were negative and 3 out of 73 lymph nodes were involved with the tumor.

**Figure 1:**
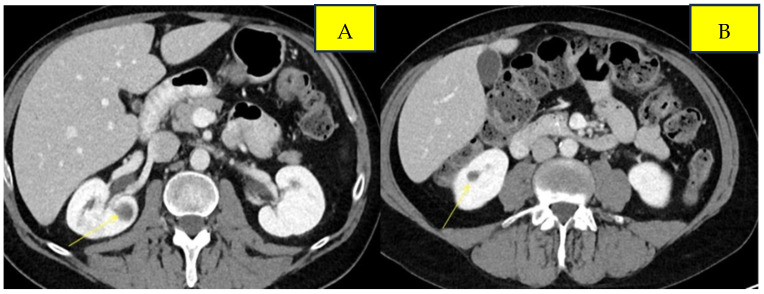
Pre-treatment imaging of bilateral renal metastases from anal squamous cell carcinoma. Panels (**A**) and (**B**) demonstrate lesions in the left and right kidneys, respectively.

**Figure 2:**
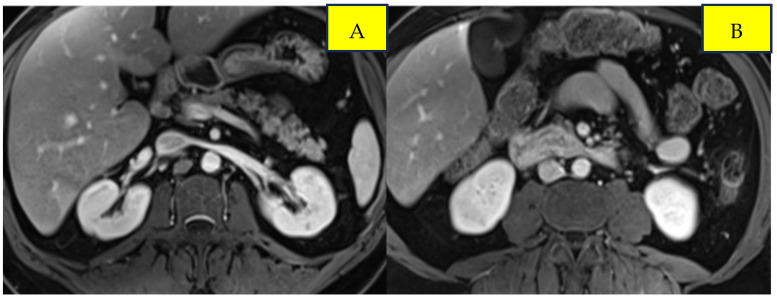
Post-treatment imaging of bilateral renal metastases from anal squamous cell carcinoma. Panels (**A**) and (**B**) demonstrate response in the left and right kidneys, respectively.

**Figure 3:**
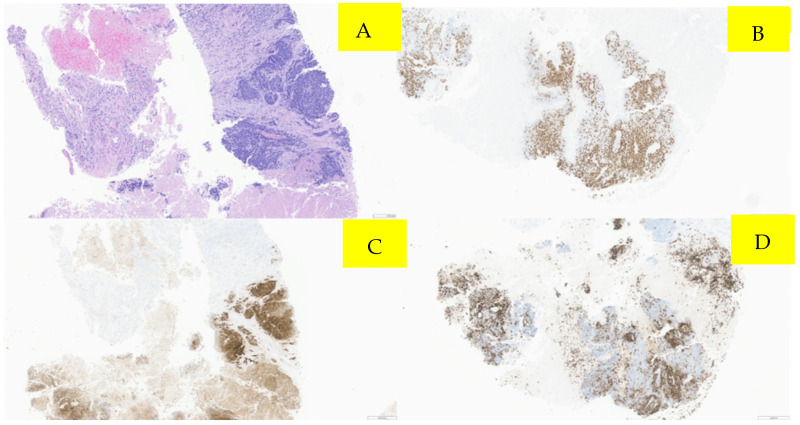
Pathology microscopic imaging, fine needle aspiration of right kidney mass. Panel (**A**) demonstrates several cores showing viable tumors with associated necrosis. (Hematoxylin and eosin staining; 40×). Panel (**B**) (40×) demonstrates immunohistochemistry showing positive p16 immunostaining, (**C**) positive p40 immunostain (40×), and (**D**) positive CK5 immunoreactivity (40×).

Surveillance scans performed six months later showed evidence of new bilateral renal hypodensities concerning metastatic disease (Figure 1A,B). A renal biopsy confirmed metastatic squamous cell carcinoma with definitive pathology findings (Figure 2). The patient was started on palliative carboplatin and paclitaxel. Restaging scans after 7 months of treatment on Carboplatin and paclitaxel showed disease progression. Treatment was therefore switched to second-line therapy with Ipilimumab/Nivolumab. Restaging scans after 5 months of treatment with Ipilimumab/Nivolumab showed a partial response to treatment and was continued on Nivolumab maintenance. At around 16 months of immunotherapy maintenance, complete response was noted and the patient continued on single-agent Nivolumab therapy every 4 weeks (Figure 3A,B).

## 3. Discussion

ASCC is an uncommon malignancy, and while locoregional metastases to liver, lungs, para-aortic nodes and skin are the most common sites [9,10], the involvement of organs such as the kidneys is exceedingly rare, with only one prior case reported in the literature [16]. Our patient represents only the second documented case of ASCC with bilateral renal metastases, highlighting an atypical pattern of disease spread.

The management of ASCC has progressed significantly over the years. The Nigro protocol, first introduced in 1974, revolutionized treatment by combining radiotherapy with 5-flurouracail (5-FU) and mitomycin C or porfiromycin [17]. Since then, chemoradiotherapy has remained the standard of care for localized disease, with the literature reporting a 5-year overall survival of approximately 60% [18], whereas systemic chemotherapy and immunotherapy have become central in managing metastatic or atypical presentations [19].

A literature search was performed in Pubmed using the terms “atypical recurrence in anal cancer, metastatic anal cancer with rare sites of recurrence, isolated renal metastases from anal cancer”. A review of previously reported cases of atypical ASCC metastases (Table 1) illustrates the heterogeneity of disease spread and clinical outcomes. Among the seven cases, four patients died from progressive disease- or treatment-related complications, while three achieved durable complete responses, underscoring the unpredictable nature of atypical ASCC progression.

Beyond reporting the sites of atypical metastases, these cases also provide valuable clinical insights into recognizing and managing unusual disease presentations. Similarly, parastomal hernia involvement has been documented [9], although the mechanism of spread to the peritoneal surface of the hernia sac remains unclear, illustrating that some metastatic patterns may be unpredictable. Another patient with cauda equina metastases [12] initially presented with perineal pain, which was misattributed to intra-abdominal recurrence and managed conservatively with morphine. Careful history and detailed neurological examination ultimately revealed spinal involvement; MRI confirmed a mass, and laminectomy resolved symptoms, with the patient remaining well at the six-month follow-up. These cases underscore the importance of maintaining a high index of suspicion, performing thorough history-taking and examinations, diagnostic imaging, and timely intervention. Early recognition and targeted management can prevent unnecessary morbidity and may improve outcomes in patients with atypical ASCC metastases.

Our patient developed bilateral renal metastases, a site reported only once previously. While most published cases of localized ASCC are managed with standard chemoradiotherapy, our patient received systemic chemotherapy (carboplatin and paclitaxel) followed by maintenance immunotherapy (Nivolumab and Ipilimumab). Platinum-based chemotherapy has demonstrated efficacy in ASCC; for instance, Meropol et al. [21] reported that induction cisplatin-based chemotherapy followed by radiotherapy achieved a complete response in 82% of patients and 61% disease-free survival at 4 years. Our patient demonstrated a favorable response, suggesting that current systemic therapies available such as platinum-containing regimens with immunotherapy may improve outcomes even in rare metastatic presentations.

This case highlights the unpredictable metastatic patterns of anal squamous cell carcinoma. To our knowledge, this is the second documented case of renal involvement. Although the exact mechanism of the metastasis in our patient remains unclear, several theories have been suggested for possible dissemination. Lymphatic spread occurs in nearly 10–15% of the cases, whereas hematogenous spread is responsible for less than 10% of the cases [22]. Metastases to the kidneys are rare, with an incidence of approximately 2.36–12.6%, and patients are usually asymptomatic [23,24]. Although the kidneys have a rich vascular supply, metastatic infiltration likely occurs via arterial embolization, and clinically evident renal metastases remain uncommon, possibly due to microenvironmental factors that make the renal parenchyma less favorable for tumor implantation [23,24]. The most common primary tumors giving rise to renal metastases include lung, colorectal, breast, head and neck, soft tissue, thyroid, and prostate cancers, with hematogenous spread being the predominant route, as reported in clinical series and autopsy studies [23,24,25].

Isolated metastatic recurrences in anal squamous cell carcinoma may behave differently from widespread disease and can be more indolent, potentially leading to better outcomes. Current National Comprehensive Cancer Network (NCCN) guidelines recommend annual imaging for surveillance [26], but this interval may not always detect early or atypical metastases, and randomized trials to optimize surveillance schedules are challenging given the rarity of this disease. When a single metastatic site is identified, local interventions such as surgery or ablation are often considered and may improve survival, as demonstrated in a single-institution retrospective study of patients with liver-limited metastatic anal cancer, in which definitive local therapy combined with systemic treatment achieved a median overall survival of over 4 years [27]. In our patient, bilateral renal involvement made local therapy unsafe, highlighting that systemic therapy alone can still yield favorable outcomes. These observations underscore the heterogeneity of disease behavior among patients with limited metastatic recurrence and the need for individualized management strategies. Our case emphasizes the importance of considering atypical metastatic sites during staging and surveillance. The favorable response to systemic chemotherapy combined with immunotherapy supports the current understanding that platinum-based regimens and immune checkpoint inhibitors can be effective even in unusual metastatic locations. Key takeaways include maintaining a high index of suspicion for rare metastatic sites, performing comprehensive imaging and biopsy for atypical lesions, and recognizing that individualized systemic therapy can yield favorable outcomes. Vigilant follow-up and tailored management remain essential for optimizing patient care in these uncommon presentations.

## Figures and Tables

**Table 1:** Summary of previously reported cases of anal squamous cell carcinoma with rare/atypical metastatic sites and treatment outcomes.

	Age	Sex	Site of Metastatic Spread	Treatment	Outcome	Reference
1	55	Male	Parastomal hernial sac	Exploratory laparotomy; colostomy takedown; bowel resections with primary anastomoses; parastomal hernia repair; abdominal wall reconstruction	Stable complete response	[9]
2	54	Female	Kidneys	Bilateral Radical Nephrectomy	The patient has remained disease-free and on dialysis for more than 8 months	[16]
3	60	Female	Spleen and lymph node near Pericardium	Splenectomy and surgical resection	Stable complete response	[20]
4	69	Female	Iris	Chemoradiotherapy	Death due to brain and medullar metastasis	[11]
5	59	Male	Cauda equina	Laminectomy	Patient was well at 6-month follow-up	[12]

## Data Availability

Data availability limited due to privacy.

## Abbreviations

The following abbreviations are used in this manuscript:

ASCC	Anal Squamous Cell Carcinoma
CRT	Chemo-radiation therapy
